# Decellularized Extracellular Matrix Scaffolds for Soft Tissue Augmentation: From Host–Scaffold Interactions to Bottlenecks in Clinical Translation

**DOI:** 10.34133/bmr.0071

**Published:** 2024-09-06

**Authors:** Yasamin Ostadi, Javad Khanali, Fatemeh A. Tehrani, Ghasem Yazdanpanah, Soheyl Bahrami, Feizollah Niazi, Hassan Niknejad

**Affiliations:** ^1^ Department of Pharmacology, School of Medicine, Shahid Beheshti University of Medical Sciences, Tehran, Iran.; ^2^ Department of Ophthalmology and Visual Sciences, Illinois Eye and Ear Infirmary, University of Illinois at Chicago, Chicago, IL, USA.; ^3^ Ludwig Boltzmann Institute for Experimental and Clinical Traumatology in AUVA Research Center, Vienna, Austria.; ^4^ Department of Plastic and Reconstructive Surgery, Shahid Beheshti University of Medical Sciences, Tehran, Iran.

## Abstract

Along with a paradigm shift in looking at soft tissue fillers from space-filling to bioactive materials, decellularized extracellular matrix (DEM) fillers have gained more attention considering their superior bioactivity. However, the complex mechanisms that govern the interaction between host tissues and DEMs have been partially understood. This review first covers the mechanisms that determine immunogenicity, angiogenesis and vasculogenesis, and recellularization and remodeling after DEM implantation into host tissue, with a particular focus on related findings from filler materials. Accordingly, the review delves into the dual role of macrophages and their M1/M2 polarization paradigm to form both constructive and destructive immune responses to DEM implants. Moreover, the contribution of macrophages in angiogenesis has been elucidated, which includes but is not limited to the secretion of angiogenic growth factors and extracellular matrix (ECM) remodeling. The findings challenge the traditional view of immune cells as solely destructive entities in biomaterials and indicate their multifaceted roles in tissue regeneration. Furthermore, the review discusses how the compositional factors of DEMs, such as the presence of growth factors and matrikines, can influence angiogenesis, cell fate, and differentiation during the recellularization process. It is also shown that the biomechanical properties of DEMs, including tissue stiffness, modulate cell responses through mechanotransduction pathways, and the structural properties of DEMs, such as scaffold porosity, impact cell–cell and cell–ECM interactions. Finally, we pointed out the current clinical applications, the bottlenecks in the clinical translation of DEM biomaterials into soft tissue fillers, as well as the naïve research areas of the field.

## Introduction

Soft tissue fillers have become essential tools in aesthetic rejuvenation, and their variety and applications in cosmetic procedures are exponentially growing [1]. The aging population, increased media coverage, consumer awareness, and new and improved filler technologies may have contributed to this expanding market demand [2]. Filler materials include biodegradable polymers (hyaluronic acid, poly-l-lactic acid, or calcium hydroxylapatite), permanent fillers (polymethylmethacrylate microspheres, hydrogel polymers, and silicone), autologous fat, and, in recent years, extracellular matrices (ECMs) [3]. The appropriate filler for each aesthetic purpose is determined by many factors, including the site of injection/implantation, volume needed, desired physiochemical and rheologic properties, cost, and invasiveness of the procedure [4].

The main advantage of ECMs over conventional fillers is their superior bioactivity, which comes from their ability to retain ECM components that are required for tissue regeneration [5]. ECM is a 3-dimensional (3D) structure containing collagen, elastin, fibronectin, laminin, and matricellular proteins [6]. In addition, numerous growth factors, cytokines, chemokines, proteases, and protease inhibitors are known to take part in ECM bioactive properties [7,8]. These structural and functional components provide structural integrity, mechanical support, and bioactive macromolecules necessary for long-term integration into surrounding tissues [9]. Therefore, decellularized extracellular matrix (DEM) scaffolds can incorporate host cells into the matrix and direct them by preserved growth factors and mechanical cues [10]. They allow angiogenesis, cell attachment, proliferation, and differentiation and are biocompatible and nonimmunogenic [10,11].

To exploit such potentials that contribute to the bioactivity and biocompatibility of DEM fillers, research is going on to assess their capabilities to replace conventional filler materials. Decellularized biomaterials derived from dermal and adipose tissues have shown promise in this regard. Decellularized adipose tissue (DAT) has shown to be a robust supporter of adipogenesis and is becoming an off-the-shelf product regularly applied to correct soft tissue defects [3]. Although the earliest applications of acellular dermal matrices (ADMs) were in severe burn injuries, the applications have been expanded to several surgical subspecialties and cosmetic procedures [10]. Other than decellularized dermal and adipose matrices, several other tissue sources, such as cartilage, intestinal submucosa, and amniotic membrane, possess unique features that make them potential filler materials, albeit less studied.

**Fig. 1.**
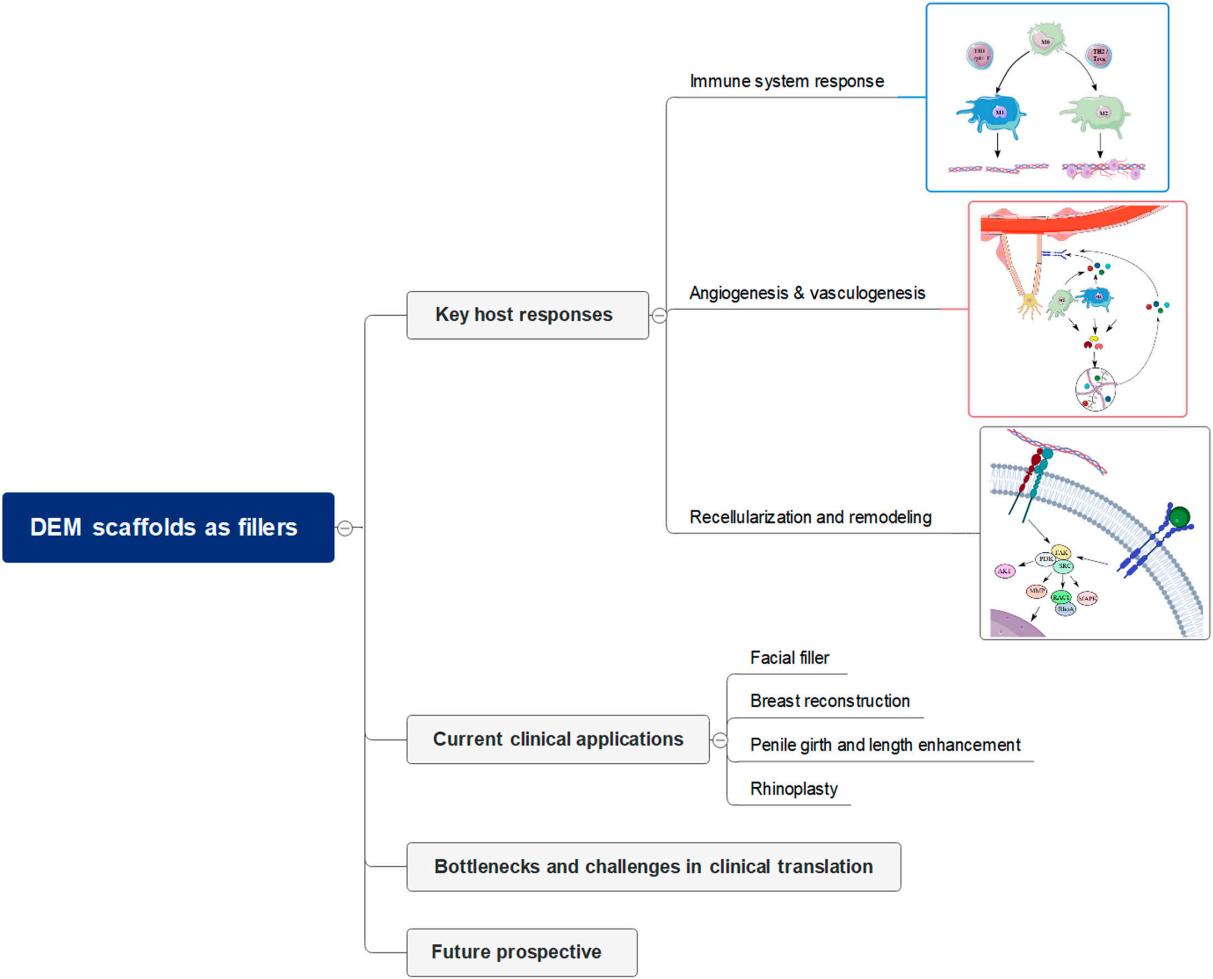
Schematic of the review structure.

Despite the application of DEM scaffolds in clinical settings for years, there is still limited knowledge of the underlying mechanisms that determine the immunological impact and bioactive capacities of the fillers in the host body. Furthermore, the realm of the applications that can be covered by DEM fillers with decisive advantages over other types of fillers is not well understood. The present review covers the latest knowledge and tackles recent developments in applying DEMs for soft tissue augmentation. Moreover, special attention is given to deciphering the mechanisms underlying host tissue responses to DEM fillers. In this review, we first discussed key host responses to DEM biomaterials including immune response, angiogenesis and vasculogenesis, and recellularization and remodeling with a particular focus on related findings from applying the biomaterials as soft tissue filler. Later, the current applications of DEMs as soft tissue fillers in clinical settings are reviewed, and the bottlenecks and challenges in translating DEM fillers into clinical applications are discussed. Last, the future direction of the field is pointed out. Figure 1 represents the structure and contents of the review.

## Key Host Responses to DEM Fillers

As mentioned, an ever-expanding array of injectable fillers with diverse mechanisms of action and composition is available to the esthetic practitioner. Each of the materials has its unique features, clinical indications, advantages, and drawbacks. Table 1 represents a comparative analysis of commercially available soft tissue fillers.

**Table 1.** Comparative Analysis of Commercially Available Soft Tissue Fillers by Material Properties and Clinical Utilization

Filler Material	Longevity (Short-lasting, Long-lasting, Permanent)	Biocompatibility	Bioactivity	Clinical Advantages	References
Decellularized Extracellular Matrices (DEMs)	Short to long-lasting; Higher volume retention than hyaluronic acid and collagen is noted in several studies.	Is proposed to be immune-privileged with no foreign body reactions.	Support stem cell proliferation, host cells infiltration, and neovascularization.	Can integrate into host tissue and support tissue regeneration.	[1–6]
			Integrate with the surrounding tissue		
			Stimulate collagen production by host fibroblasts.		
			Incorporates all structural (e.g., collagen, GAGs, elastin) and functional (e.g., growth factors) components of ECM.		
Collagen fillers	Short-lasting; Advantage: long-term adverse complications are not an issue.	There are cases of filler-induced embolism and allergies.	Support neovascularization to a lesser extent than DEMs. Do not integrate with the surrounding tissue. Stimulate collagen production by host fibroblasts.	Easy to use The oldest FDA-approved dermal fillers (their safety is well-established)	[1,3,5,7]
	Disadvantage: repeated injections are needed to maintain their volume.	Antigenicity is concerned and skin test may be required.			
Hyaluronic acid fillers	Short-lasting; Advantage: long-term adverse complications are not an issue.	There are cases of filler-induced embolism.	Do not support cell infiltration and neovascularization. Do not integrate with the surrounding tissue. Stimulate collagen production by host fibroblasts.	Reversible (by injection of hyaluronidase)	[1,3,4,8,9]
				Easy to use	
	Disadvantage: repeated injections are needed to maintain their volume.	Is more frequently involved in vascular occlusions than other fillers.		Low viscosity that enables improving the appearance of fine lines	
				Amenable to molding	
Biodegradable synthetic polymers (e.g., calcium hydroxyl apatite, Poly-L-lactic acid (PLLA))	Long-lasting; Advantage: Augment tissue volume for up to several years. Disadvantage: can lead to immunologic complications.	Foreign body reactions may be a concern including the presence of giant cells, capsule formation, and granuloma formation.	Stimulate collagen production by host fibroblasts.	Positioned best for deeper contour deficiencies and adding volume	[1,2,8–10]
		There is a risk of initial failure with infection.			
Non-biodegradable synthetic polymers (e.g., polymethylmethacrylate, silicone)	Permanent; Advantage: No need for repeated procedures to maintain volume.	Foreign body reactions are common including the presence of giant cells, capsule formation, and granuloma formation. There is a risk of initial failure with infection.	Stimulate collagen production by host fibroblasts.	Applicable for patients with more substantial volume loss	[1,2,8–10]
	Disadvantage: Can lead to immunologic complications.				
	Invasive procedures may be needed for filler removal.				

Filler materials with a positive interaction with host tissues would show a more favorable integration profile and become more durable. Some injectable fillers such as hyaluronic acids and collagens are widely applied in cosmetic procedures; however, these materials cannot integrate with the surrounding tissues, and thus, they are rapidly absorbed in vivo [12]. Furthermore, the positive interaction minimizes the risk of adverse reactions, such as acute rejection, which can compromise the effectiveness of the filler and lead to undesirable outcomes.

ECM acts as the microenvironment in which cells attach and interact; thereby, ECM regulates cell dynamics and behaviors and maintains tissue-specific functions and phenotypes. Similarly, DEM scaffolds allow the seeding and proliferation of specific cells while being degraded and replaced with new tissue [13]. The outcome of DEM fillers can vary from tissue regeneration and augmentation to inflammation, fibrotic tissue formation, and necrotic processes. The difference is determined by the host tissue response to DEM implants and involves a complex interplay of biochemical and biomechanical processes [14]. In this section, host responses to DEMs are categorized into immune system response, angiogenesis and vasculogenesis, and recellularization and remodeling. However, it should be noted that all host responses to DEMs cannot be assigned to 1 of these 3 categories. Furthermore, a holistic view of crosstalk and interactions that exist between these host responses is needed to provide an overview of scaffold–host interactions.

### Immune system response

Biomaterials that are used for soft tissue filling can be broadly categorized into synthetic and natural materials. It is generally presumed that natural materials are less immunogenic and more biocompatible. Synthetic materials are manufactured of polymers, chemicals, metals, or other synthetically derived substrates. These materials, especially those that are nondegradable, are associated with a pro-inflammatory host response [15]. Initial inflammatory response to synthetic implanted materials may progress to an unresolved chronic inflammation, which persists as long as the implant remains, called the host foreign body reaction [16]. This terminal state is determined by the prolonged presence of activated macrophages and foreign body giant cells at the tissue–biomaterial boundary and the formation of a dense fibrous capsule. The severity of the foreign body reaction is commonly assessed by the presence of foreign body giant cells and the thickness of the fibrous capsule [16].

On the other hand, natural biomaterials are processed from whole ECMs (e.g., DEM scaffolds) or purified individual ECM components (e.g., collagen, laminin, fibronectin, and silk). DEM scaffolds, as an example of natural biomaterials, are prepared by methods that ideally remove most of the cellular remnants and major histocompatibility complex (MHC) antigens and conserve ECM structures, which makes the host immune system recognize them as self-components [17,18]. Removing MHC complexes, donor antigen-presenting cells, cell interactions, and presumably extracellular vesicles in the decellularization process would restrict allorecognition and privilege implant from acute rejection [16,18].

The most relevant sources of immunogenicity in the context of DEM implants are the damage-associated molecular patterns (DAMPs) [19]. DAMPs are danger signals released upon cell or ECM damage during tissue processing, decellularization, and implantation, which signal stress or uncontrolled cell death [18,19]. In the context of DEM scaffolds, nuclear and mitochondrial DNA released by perturbation in the arrangement of cell nucleic acids, reactive oxygen species produced due to reperfusion imperfection, and fragmented ECM components resulting from harsh decellularization strategies are among the most prominent DAMPs [18].

Another source of immunogenicity in DEM biomaterials is incomplete decellularization [20]. Residual MHC antigens, DNA fragments, and α-Gal (in xenograft implants) are the most renowned antigens that are intrinsically linked to immunogenicity [21]. The immunostaining of DEM structures for MHC complexes showed that the absence of MHC complexes within the matrix would remove the allo-antigenicity from DEMs, and the implants would undergo remodeling as indicated by host cell infiltration and neovascularization [22,23]. Conversely, DEMs with residual MHC complexes despite decellularization showed continued proliferation of CD4^+^ T cells in vitro and eventual implant degradation in vivo [24]. On the other hand, the attempt to completely remove cellular materials necessitates a robust decellularization process, which may damage ECM structures and consequently have detrimental effects on the matrix’s regenerative properties and itself be a source of immunogenicity [25]. The generation of fragmented fibronectin, heparin sulfate, and hyaluronic acid during the DEM decellularization process can act as DAMPs and affect the host’s immune response after implantation [26,27]. In this regard, it is shown that uterus matrices, which are produced by stronger decellularization detergent, released more ECM-related DAMPs and resulted in early and persistent pro-inflammatory cytokine response [27]. There is still an ongoing debate on the optimum decellularization method to decrease DEM immunogenicity along with preserving biomaterial bioactivity.

**Fig. 2.**
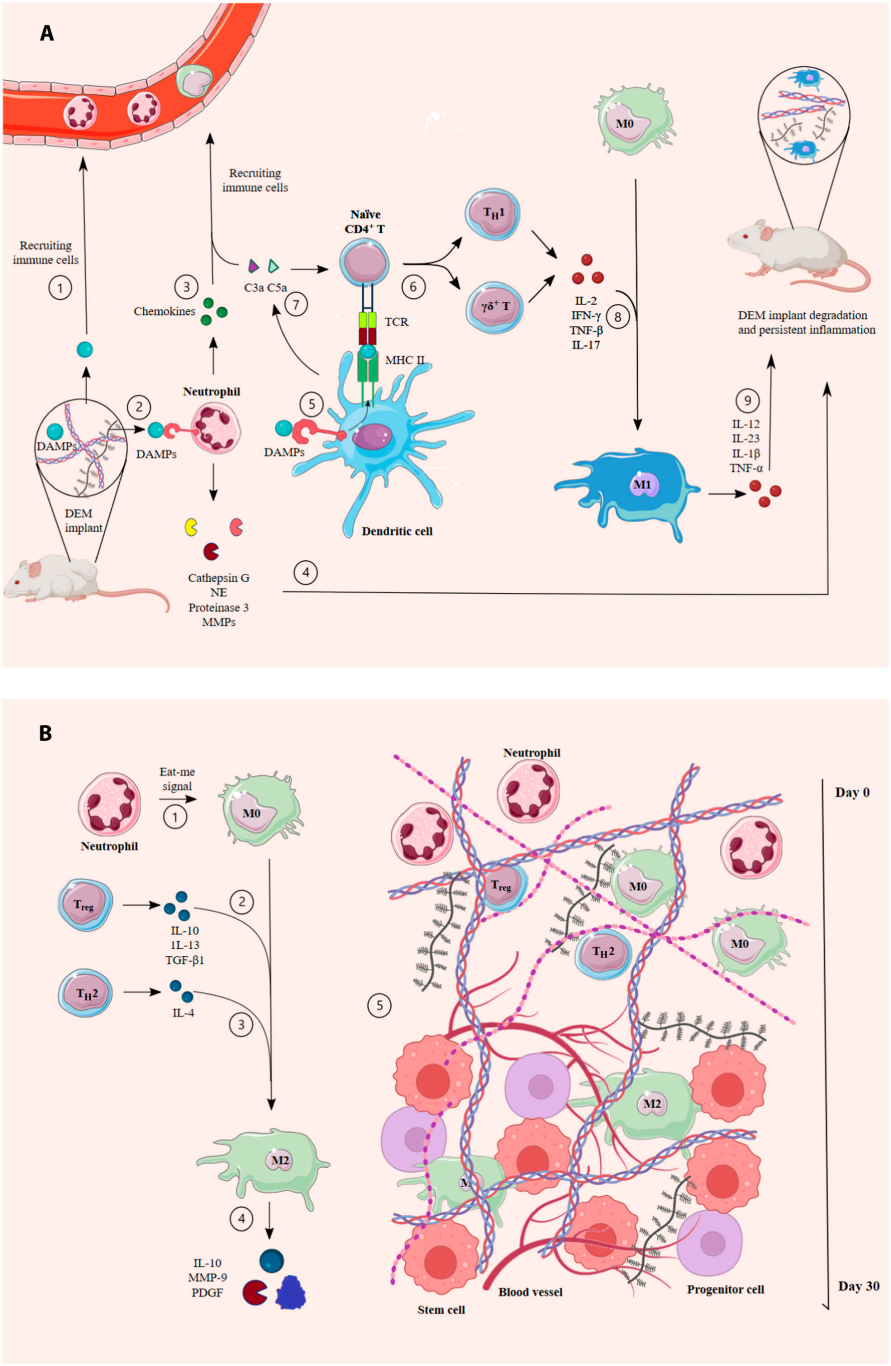
Immune cell recruitment and macrophage polarization in response to DEMs during: (A) Destructive immune response to DEMs. (1) Damage-associated molecular patterns (DAMPs), which are produced during the decellularization process, recruit neutrophils to the implantation site. (2) DAMPs are recognized by neutrophils through pattern recognition receptors. (3) Subsequent to DAMP recognition, neutrophils release chemokines that recruit other immune cells such as monocytes and lymphocytes to the implant site. (4) Neutrophils also produce cathepsin G, neutrophil elastase (NE), proteinase 3, and matrix metalloproteinases (MMPs), which collectively contribute to the digestion of the extracellular matrix (ECM). (5) Antigen-presenting cells including tissue dendritic cells are other cells that detect DAMPs by their pattern recognition receptors and represent the antigens via the MHC class II complex to native CD4^+^ T cells. (6) Dendritic cells promote the transformation of native CD4^+^ T cells into T helper 1 (T_H_1) and γδ T cells. (7) Dendritic cells also secrete C3a and C5a, which further induce CD4^+^ T cell transformation and immune cell recruitment to the implant site. (8) T_H_1 and γδ T cells secrete IL-2, interferon-γ (IFN-γ), tumor necrosis factor-β (TNF-β), and IL-17 cytokines that promote macrophage polarization toward the M1 phenotype (9). The macrophage phenotype ultimately causes persistent inflammation and DEM degradation by secreting inflammatory cytokines. (B) Constructive immune response to DEMs. (1) In the absence of DAMPs, recruited neutrophils emit “eat-me” signals, which trigger the macrophages to polarize into the M2 phenotype. (2 and 3) In parallel, T_H_2 cells and regulatory T cells contribute to M2 macrophage polarization by secreting IL-4, IL-10, IL-13, and TGF-β1. (4) The M2 macrophages promote the resolution phase of inflammation by the secretion of regenerative and anti-inflammatory cytokines and enzymes. (5) Following a 30-day constructive immune process, stem/progenitor cells are recruited, new blood vessels are formed, and the implant is recellularized.

As shown in Fig. 2, the immune response to DEM biomaterials can be constructive or destructive for the implant, which is based on the level of immune system activation and allorecognition. The major determinants of constructive immune response to DEM scaffolds are the temporal innate immune activation and the anti-inflammatory macrophage phenotype. This kind of immune response to bioscaffolds would reduce local inflammation and lead to constructive crosstalk between biomaterial and stem and progenitor cells (Fig. 2) [15].

Many immune cell types conduct the process of immune response to DEM biomaterials. The first cells that contribute to the immune response to implants and appear at the implant site are neutrophils. The initial recruitment of neutrophils is mediated by recognizing DAMPs by their pattern recognition receptors [20]. The recruitment of neutrophils would be further amplified via CXCL8 chemokines and leukotriene B4, which are produced by neutrophils and attract more distant neutrophils. When neutrophils become activated, they mediate matrix reprogramming, angiogenesis, and regeneration and consequently recruit other immune cells such as monocytes and lymphocytes. It is shown that undisturbed neutrophil function is the key to initiating and regulating downstream immune responses to biomaterials, such as macrophage infiltration and inflammation levels. Early depletion of neutrophils results in a poor retention rate of fat grafts, whereas up-regulated neutrophils increase the inflammation and reactive oxygen species level, leading to tissue damage [28]. During the constructive immune response to DEMs, neutrophils will eventually undergo apoptosis and give off eat-me signals to attract macrophages and be engulfed, phagocytosed, and digested [29].

The mainstay of the constructive immune response to DEM implants is the anti-inflammatory M2 macrophages. The M2 macrophage phenotype is required for tissue repair and regeneration in biological ECM scaffolds. M2 macrophage phenotype supports tissue repair and reconstruction by anti-inflammatory cytokine production, stem/progenitor cell recruitment, cell replacement, and matrix remodeling. The role of M2 macrophage response to biological scaffolds in tissue restoration and regeneration was validated clinically in biopsies from large muscle defects treated with DEM [30,31]. Studies demonstrated that when macrophage progenitors were depleted in animal models, ECM scaffold remodeling was severely impaired [32]. Examining the adipose tissue regeneration model demonstrated a transition of cytokines from pro-inflammatory to anti-inflammatory concurrent with the transition of macrophage phenotype from M1 to M2, indicating the role of M2 macrophages in anti-inflammatory cytokine production [33]. It is also shown that depletion of M2 macrophages in fat grafts would result in weak stem cell recruitment and a poor volume retention rate [34]. Furthermore, studies on DAT revealed that macrophage-derived factors, such as monocyte chemotactic protein-1, induced adipogenesis by recruiting macrophages and bone marrow-derived precursor cells in the first 2 days after DAT implantation into host tissue [35].

The major driver of M2 polarization in response to biological scaffolds is shown to be the type-2 T helper (T_H_2) cells. Preclinical studies showed that a local T_H_2 response is formed and guides interleukin-4 (IL-4)-dependent macrophage polarization and promotes the M2 macrophage phenotype [36]. A study in Rag1^−/−^ mice (which lacks mature B and T cells) showed that a pro-regenerative microenvironment for biomaterials requires T_H_2 cells. In the study, mice were repopulated with either wild-type CD4^+^ T cells or Rictor^−/−^ CD4^+^ T cells (which could not polarize to T_H_2). Accordingly, the Rictor^−/−^ CD4^+^ T cell group showed diminished M2 macrophage quantity, indicating the role of T_H_2 cells in M2 macrophage polarization [36]. Other cells that have a fundamental role in promoting M2 macrophage polarization are regulatory T (T_reg_), which exerts the function through secreting IL-10, IL-13, and transforming growth factor-β (TGF-β1) cytokines [37]. Studies on DAT for de novo adipogenesis and soft tissue augmentation revealed that methoxy polyethylene glycol-modified DAT showed markedly lower immunogenicity and higher adipogenesis than unmodified DAT, due to increased T_reg_ cell numbers, and in turn enhanced the M2/M1 macrophage ratio [37]. Giving off eat-me signals by neutrophils during a constructive immune response is another trigger for macrophages to switch their phenotype to anti-inflammatory M2. The signal, when emitted by dying neutrophils, leads to subsequent engulfment and phagocytosis of the cells by macrophages and stimulates them to develop the M2 phenotype [29].

Successful ECM-derived biomaterial can effectively induce macrophages to polarize from M1 to M2 within 7 to 14 days [38,39], and subsequently, the resolution phase of inflammation begins after 15 days of transplanting DEMs [37,40]. During the resolution phase, the number of neutrophils decreases, M2 macrophages outnumber the M1 phenotype, and anti-inflammatory cytokines are secreted [38,39]. Studies on DAT material also revealed that implants were initially infiltrated with macrophages, which decrease in number as adipocyte number increases [41]. Accordingly, a study reported a great number of leukocytes and macrophages in the DAT implant 2 weeks after implantation, which decreased after 4 and 8 weeks [12]. Another study showed that macrophages infiltrated tissue after 3 days, peaked at day 7, and significantly decreased thereafter. The M2/M1 ratio of macrophages also significantly increased from day 3 to 7, and M2 macrophages became the predominant macrophage population in 42 days [33].

In contrast to T_H_2/T_reg_ and M2 macrophage-mediated constructive immune response, a destructive immune reaction in response to excessive DAMP release and residual antigens can be developed in response to implants (Fig. 2). In the absence of cytotoxic T cell activity due to the acellular nature of the implants, the involved immune mechanisms are mediated by macrophages and neutrophils, which are effector cells of the innate immune system, with the assistance of helper T cells [42]. The compartments of the immune response against DEM implants also depend on the source of ECM. For instance, α-Gal epitope and anti-Gal antibodies are prominent immune reaction compartments in xenografts but have no functional relevance in allografts [42]. The cell phenotypes that take part in destructive immune reactions against DEMs include M1 macrophages fusing to form foreign body giant cells and type-1 T helper (T_H_1) cells, IL-17-producing γδ^+^ T cells, and senescent stromal cells in the fibrotic capsules [43]. It is shown that a sustainable number of neutrophils may increase the M1 macrophage recruitment, resulting in its fusion with foreign body giant cells and ultimately causing persistent inflammation [44]. The remaining antigens after the decellularization process such as major and minor histocompatibility antigens can activate cellular immune response. Moreover, DAMP-mediated complement cascade activation releases C3a and C5a components, which induce T_H_1 and T_H_17 polarization in CD4^+^ T cells [18]. Accordingly, the key to escaping destructive innate and adaptive immune responses is to inhibit excessive DAMP release mainly through preservation of the ECM structure during decellularization and a robust antigen removal process [18]. Figure 2 represents a schematic of the described host immune system response to DEM biomaterials.

Recent studies have introduced novel ideas regarding biomaterial-associated macrophage populations. First, it is shown that the classical M1/M2 polarization paradigm does not ideally justify macrophage phenotypes in biomaterials. A study that implanted DAT to augment soft tissue demonstrated infiltration of CD4^+^ T cells into the implants and enhanced hybrid M1/M2 macrophage responses in both preclinical and clinical settings [45]. Hybrid macrophages may represent a transition state between M1 and M2 phenotypes or are the outcome of simultaneous M1/M2 cytokine signals. Second, it is shown that the sources of tissues from which DEM scaffolds are derived have a direct impact on the phenotype of infiltrated macrophages [46]. For instance, macrophages exposed to small intestinal submucosa, urinary bladder matrix, brain ECM, esophageal ECM, and colonic ECM express a predominant M2 macrophage phenotype, while macrophages exposed to dermal ECM resulted in a predominant M1 phenotype. It is perceived that matrix-bound nanovesicles are the directors of this phenomenon [47]. The nanovesicles and their associated miRNA cargo recapitulate the effects of the ECM bioscaffold from which they are derived on macrophages and play a significant role in determining the effects of ECM bioscaffolds on macrophage phenotype. Further studies are needed to delineate the fate of macrophage subsets and their relevant role in tissue repair or inflammation after DEM implantation.

### Angiogenesis and vasculogenesis

After implantation of the DEM structures in vivo, the formation of a functional vascular network is another host response that is mandatory for transferring oxygen and nutrients to implants. Without vascularization, oxygen and nutrients can only diffuse to 200-μm depth in the implanted grafts in vivo [48]. Therefore, the creation of mature functional blood vessels is a critical challenge in the field of tissue engineering and regenerative medicine. The formation of new blood vessels involves both angiogenesis and vasculogenesis [48]. Angiogenesis, a process by which new blood vessels sprout or directly split from preexisting vascular plexuses, is the primary mechanism responsible for neovascularization in response to DEM biomaterials. In the angiogenesis process, blood vessel endothelium specializes in tip and stalk cells to expand the vascular network by sprouting. The tip cells are specialized to extend filopodia and detect chemotactic growth factor gradients, while the stalk cells form a lumen to transport blood [49]. On the other hand, vasculogenesis is a process by which new blood vessels are formed by individual endothelial cells (ECs) de novo. However, the importance of this process in the vascularization of DEM implants is not fully documented and has remained obscured. Through the vasculogenesis process, endothelial progenitor cells (EPCs), which are derived from bone marrow and/or resident in the vascular wall, contribute to neovascularization. At neovascularization sites, EPCs differentiate into ECs and become incorporated into the endothelial lining [48].

ECMs are assumed to be suitable angiogenic materials since they provide essential components for driving microvessels including vascular endothelial growth factors (VEGFs), fibroblast growth factors (FGFs), platelet-derived growth factors (PDGFs), and angiopoietins. Some of these factors are necessary for angiogenesis, while others tune this process. VEGF family contains several distinct proteins that are crucial regulators of angiogenesis. The growth factor induces proliferation, sprouting, and tube formation of ECs. PDGF has a significant role in angiogenesis. Secreted PDGF regulates angiogenesis through pericyte recruitment and stabilization of neovessels in both autocrine and paracrine manner. Knockouts of PDGF-B and PDGF receptor-β (PDGFR-β) caused vascular dysfunction, leading to vascular leakage and abnormal junctions [50]. Among the FGF family, FGF-1 and FGF-2 have a remarkable role in angiogenesis. FGFs bind to ligand-stimulable tyrosine kinase receptors and induce the enzymatic activity of these receptors. These biological responses eventually lead to cellular activities including migration, proliferation, and differentiation by activating tip and stalk cells, which subsequently migrate and elongate [51]. Angiopoietins are a family of vascular growth factors that have a role in the stabilization and maturation of blood vessels. Angiopoietin-1 (Ang-1) interacts with the ECM and induces cell adhesion and motility [50]. Although acellular matrix products contain significant growth factors, their concentration decreases through decellularization. It is shown that only 52% and 30% of VEGF and FGF-2 were maintained in decellularized vessel scaffolds, respectively. This indicates that further modifications are needed to achieve a higher growth factor retention rate during the decellularization process [52].

The superior angiogenic capability of DEMs over other natural fillers has been demonstrated in several studies. A study described the in vivo function of DAT hydrogels compared to Juvederm hyaluronic acid filler for treating subcutaneous volume deficits. The study showed that DAT hydrogel combined with either adipose-derived stem cells (ASCs) or the transglutaminase cross-linker has superior neovascularization in vivo [53]. Allograft DAT can also retain VEGF and FGF angiogenic factors even at higher concentrations than native adipose tissue and demonstrate robust angiogenesis after subcutaneous implantation [41,54]. Other decellularized tissue matrices have also shown such proangiogenic properties. A study showed that decellularized small intestine submucosa (SIS) gel can sustainably release VEGF and FGF-2, and no initial burst release was observed. The SIS gel evoked more powerful neovascularization than collagen type I gel, which is determined by tube formation experiments in human umbilical vein endothelial cells (HUVECs) and the mouse aortic ring assay. The up-regulated expression of key genes in angiogenesis such as kinase insert domain receptor (KDR), Notch1, and Ang2 was demonstrated in the epithelial cells of the human umbilical vein when they were seeded on the SIS gel and confirmed the presence of angiogenic factors in the biomaterial. The SIS gel also significantly increased neovascularization compared to the collagen type I gel in vivo [55]. As another angiogenic DEM, it is shown that adding ADM to fat graft reduces fat necrosis and liquefaction and increases CD34 expression in fat mass (a biomarker of vascular cells and macrophages) by promoting the infiltration of inflammatory cells and the regeneration of blood vessels [56].

In addition to the rich array of angiogenic growth factors and matricellular proteins in DEMs, it is now evident that the immune system’s response to DEMs plays an indispensable role in angiogenesis. Studies have shown that adding zymosan-A, a sterile inflammatory agent, to silicone tissue engineering chambers containing Matrigel, a basement membrane-rich ECM, can promote neovascularization in vivo. Considering that zymosan interacts with pattern recognition receptors on immune cells and increases their infiltration into the constructs, these results highlight the role of immune cells in the angiogenesis of ECM implants [57]. Macrophages, which are key determiners of immune response to DEM implants, play a pivotal role in angiogenesis induction in DEM structures. When macrophages were depleted in Matrigel-containing tissue engineering chambers using clodronate liposomes, there was minimal new vascular tissue development [58]. In addition, early activation of macrophages using a macrophage colony-stimulating factor allowed better angiogenesis in fat grafts, showing that macrophages were essential for tissue revascularization [34].

The primary role of macrophages in angiogenesis is thought to be secreting angiogenic growth factors [59]. Macrophages also influence angiogenesis through ECM remodeling and dictating controlled ECM degradation, which paves the way for migrating cells. Moreover, enzymatic degradation of some ECM molecules such as hyaluronic acid or fibrin by macrophages produces degradation products that have angiogenic properties [60]. Furthermore, ECM enzymatic degradation by macrophages releases several angiogenic growth factors that are trapped in the ECM structure. Likewise, ECM remodeling alters the physical forces acting on ECs and influences their responsiveness to pro-angiogenic growth factors.

Another potential mechanism by which macrophages influence angiogenesis is through transdifferentiation and vascular mimicry. It has been suggested that macrophages have the ability to transdifferentiate into ECs and express EC-associated markers such as PECAM-1 and VE-Cadherin and form vascular mimicry channels [61]. In addition, macrophages promote the fusion of neighboring tip cells to add new circuits to the existing vessel network. In other words, macrophages act as cellular chaperones for the formation of vascular networks in the angiogenesis process [49]. Regarding the phenotype of macrophages that contribute to angiogenesis, it is shown that the pro-angiogenic abilities of macrophages blur the boundaries of M1 pro-inflammatory and M2 anti-inflammatory traditionally defined phenotypes, and coordinated contributions from both M1 and M2 macrophages are required for angiogenesis and scaffold vascularization [59].

**Fig. 3.**
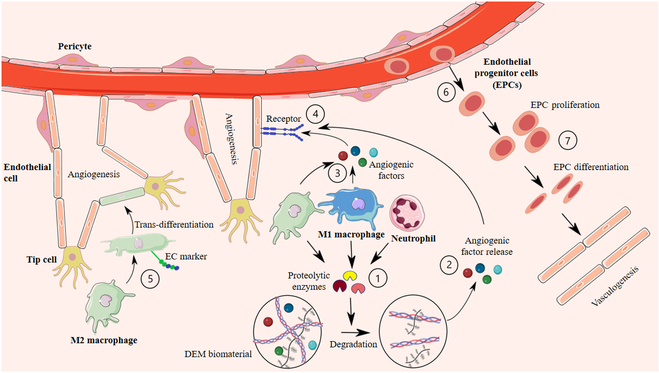
Mechanisms of angiogenesis and vasculogenesis in DEM biomaterials. (1) M2 and M1 macrophages, as well as neutrophils, secrete proteolytic enzymes that facilitate the digestion of extracellular matrix (DEM) biomaterials. (2) This enzymatic activity results in the release of angiogenic factors from the DEM. (3) Macrophages also produce angiogenic factors, including vascular endothelial growth factor (VEGF), platelet-derived growth factor (PDGF), angiopoietin, and fibroblast growth factor-2 (FGF-2). (4) These angiogenic factors interact with their endothelial receptors, triggering signaling cascades that promote angiogenesis. (5) Notably, M2 macrophages possess the ability to undergo endothelial transdifferentiation, further enhancing their capacity to support neovascularization. (6) During vasculogenesis, endothelial progenitor cells (EPCs), which are derived from bone marrow or resident within the vascular wall, play a critical role. Upon appropriate stimuli, EPCs detach from the vessel wall and migrate into the surrounding tissue. (7) Stimulated EPCs contribute to the formation of new blood vessels through their differentiation into mature endothelial cells and subsequent assembly into functional vasculature. The process of vasculogenesis supplements angiogenesis and promotes the development of a well-organized vascular network within the tissue.

Neutrophils are another type of immune cells contributing to angiogenesis in DEM structures. They facilitate ECM degradation and remodeling by secreting specific neutrophil serine proteases, including proteinase 3, neutrophil elastase, cathepsin G, and matrix metalloproteinases (MMPs). Therefore, neutrophils promote the release of DEM angiogenic factors and support migrating ECs [20]. It is shown that early depletion of neutrophils in autologous fat grafts results in incompetent angiogenesis, accompanied by significantly decreased MMP-9 level [28]. However, the impact of neutrophils on angiogenesis in implanted biomaterial structures has yet to be robustly documented. Figure 3 summarizes the proposed mechanisms for angiogenesis in DEM biomaterials.

Although DEMs are naturally angiogenic materials, there is a high demand for achieving an intact and functional vascular structure within these biomaterials to ensure their integration with the host tissues. In this context, studies have shown that adding growth factors, extracellular vesicles, and stem cells to the DEM structure can further enhance the biomaterial’s angiogenic capacities. Research has demonstrated that scaffolds loaded with angiogenic growth factors, such as FGF-2 and VEGF, stimulate blood vessel infiltration [62–64]. Heparinized DEMs, capable of encapsulating and sustainably releasing significantly higher amounts of FGF-2 and VEGF, formed well-maintained, vascularized, and recellularized tissue compared to control DEMs. Extracellular vesicles were also employed to improve the angiogenesis of decellularized tissues. It is shown that adipose tissue-derived extracellular vesicles are mediators for angiogenesis and adipogenesis. The extracted extracellular vesicles, loaded into DAT hydrogel, enhanced angiogenesis and adipogenesis; therefore, they increased tissue volume at 4 and 8 weeks postoperatively in the severe combined immunodeficient (SCID) mice model [65]. Since some DEMs such as ADM have a compact structure, they can show delayed post-implantation vascularization. It was shown that the human ASCs cocultured with ADM sponges promote proliferation, tube formation, and migration of ECs in vitro [66]. The addition of ASCs to ADM constructs also increased the expression of genes associated with inflammatory regulation, angiogenesis, and stemness through paracrine signaling [67]. After implantation of the ASC-loaded filler in vivo, the vascularization was faster, and more adipose tissue was formed [68]. Moreover, ASC seeding on DAT bioscaffolds showed a significantly enhanced in vivo angiogenesis and adipogenesis [69].

### Recellularization and remodeling

The third host reaction that drives DEM integration into host tissue is the recellularization of biomaterial with host-derived cells in the implant site. DEM scaffolds consist of the same components as the 3D framework of ECM. Furthermore, after decellularization, the physicochemical cues and biological roles of ECM can be preserved, providing mechanical and biological support for subsequent cell seeding. Therefore, host-integrated DEMs can be developed after the implantation of the biomaterial in the tissue-filling procedures [6].

ECM is composed of a tissue-specific proportion and organization of collagens, elastin, cell adhesive glycoproteins, proteoglycans, and an array of growth factors and matricellular proteins. The tissue specificity imparts unique properties that act in concert in regulating the ultimate phenotype and function of infiltrated cells [13]. For instance, adipose tissue ECM-based products had a great potential to promote de novo adipogenesis, which is promising for replacing autologous fat transfer [11]. It is believed that DATs can retain the cytokine composition of adipose tissue and support ASC proliferation and adipogenesis. A study that produced DAT matrix powder suspended in physiological saline showed gradual degradation of the matrix and in vivo adipogenesis when the material was subcutaneously injected into mice [70]. Other studies applying DAT suspension also repeated the promising results, and DAT showed long-term soft tissue volume expansion, formation of adipose tissue palpably consistent with subcutaneous adipose, and no severe adverse events [41,54]. In all the studies, ASCs that were seeded onto the allograft adipose matrix underwent adipogenesis with no external adipogenic media stimuli [12], which indicates that DAT preserved a similar composition and cytokine profile with the native adipose ECM [71].

**Fig. 4.**
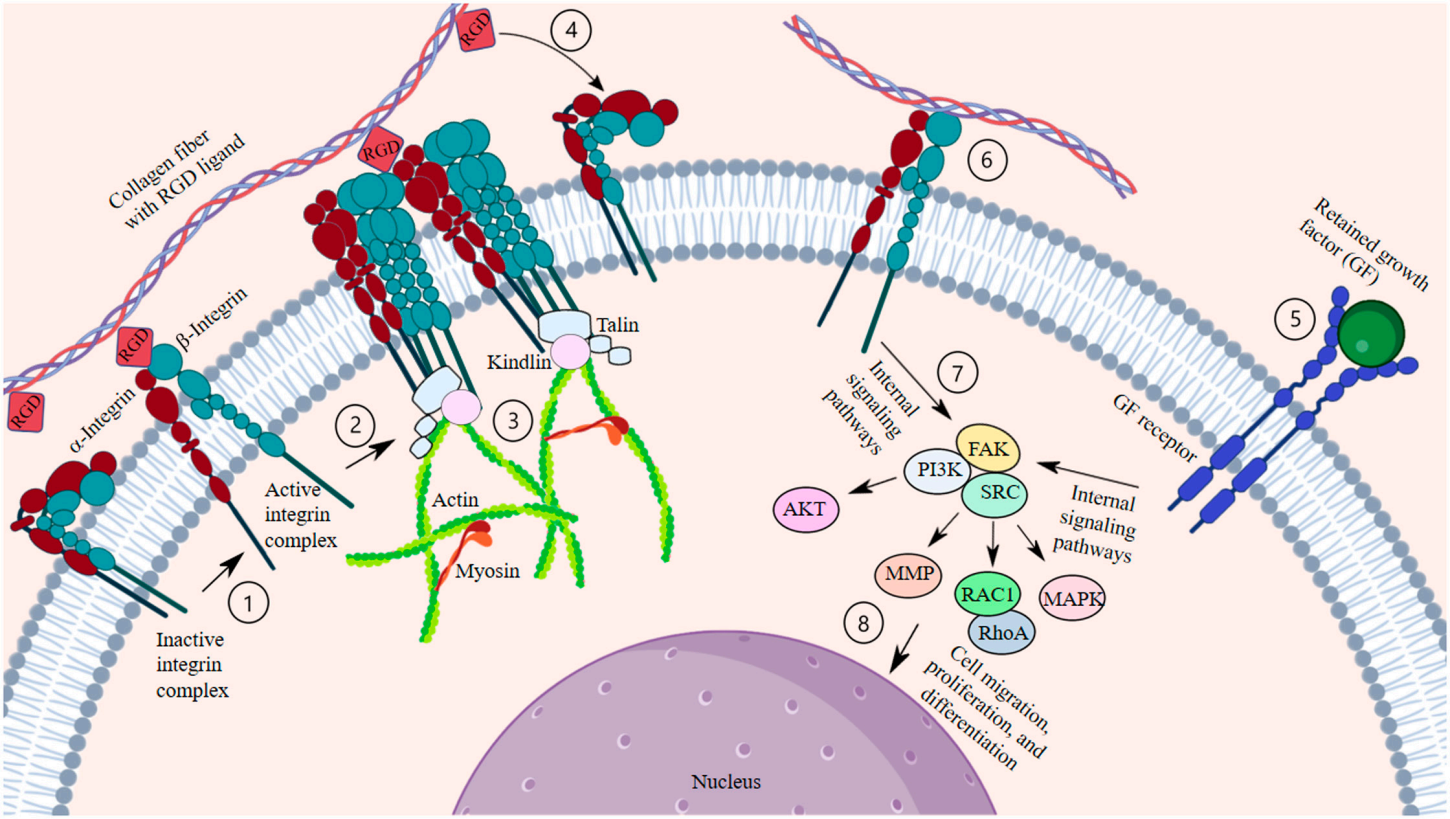
Compositional and biomechanical properties of DEMs that influence reseeded cell behavior. DEM scaffolds interact with stem/progenitor cells through various mechanisms. (1) Cell-interacting cues such as RGD peptides can bind with integrin receptors. Upon binding to ECM molecules, an extended activated integrin complex is formed, and the transmembrane helices dissociate. (2) Then, the integrin subunits can homo-oligomerize to dimers (α-subunit) or trimers (β-subunit), which can aggregate with other integrins or other proteins and form highly complex focal adhesions. (3) In the intracellular site, the complex interacts with proteins such as talin, kindlin, actin, and myosin filaments. RGD-containing ligands can bind to both the initial homodimeric state and the focal adhesion-ECM complex. (4) Recurrent cell spreading in the direction of the RGD gradient leads to the formation of new adhesion complexes and detachment of the old ones, which promotes cell migration. (5) Growth factors that are retained in DEM structure act on cells by their cell surface receptors. (6) Mechanical forces exerted on the scaffolds are sensed by integrin receptors, thereby influencing cell responses through mechanotransduction pathways. (7) Both growth factor receptors and integrins activate internal signaling pathways through their end domains. (8) The pathways determine cell differentiation, proliferation, and migration.

Despite the proven capability of decellularized scaffolds in tissue engineering, the molecular mechanisms underlying stem and host cell interactions with decellularized scaffolds remained unclear [72]. However, it is shown that compositional, biomechanical, and structural features of DEMs influence infiltrated stem or progenitor cell behavior [13]. ECM can majorly preserve its compositional factors to support its biological role after decellularization. As shown in Fig. 4, DEM scaffolds and stem/progenitor cells can interact with each other through retained growth factors [13]. Such growth factors may have a crucial role in determining cell fate and differentiation through the recellularization process. A study compared the construction of cartilage tissue using Wharton’s jelly ECM or articular cartilage ECM, given that Wharton’s jelly contains some chondrogenic growth factors, such as insulin-like growth factor I and TGF-β. Results confirmed that DEM derived from Wharton’s jelly contains more chondrogenic growth factors than cartilage, and the Wharton’s jelly scaffold was superior in enhancing chondrogenesis, as indicated by higher expression of collagen II and aggrecan mRNA expression [73]. As another compositional factor, matrikines, which are ECM degradation products generated through the proteolytic action of matrix MMPs and ADAMs (a disintegrin and metalloproteinase) family members, can modulate recruiting stem/progenitor cells, cell adhesion, migration, and differentiation [74,75]. Examples of these matrikines include the Arg-Gly-Asp (RGD) peptide derived from fibronectin and collagen degradation, which can promote cell adhesion and migration [76]. RGD peptides promote migration by binding to integrin receptors. Integrins are heterodimers that couple ECM proteins to intracellular signalings and cytoskeletal complexes. When the bond is formed, other integrins are recruited, and actin filaments are assembled and aggregated by myosin contraction. Actin-dependent spreading, which drives ligated integrin clusters outward, followed by myosin-mediated retraction, which draws integrin clusters inward, leads to cell migration [77]. In this regard, the effect of covalently bounded RGD peptide gradients in polyethylene glycol diacrylate hydrogels on cell behavior was studied. Results demonstrate that cells recognize and tend to migrate in the direction of the RGD gradient. The speed of cell migration in the hydrogel with RGD gradient was higher than that of hydrogels with a uniform distribution of RGD and increased by raising the RGD gradient steepness [78]. It is also demonstrated that RGD modification of polyethylene glycol hydrogel significantly increased EPC adhesion and viability in the biomaterial. The average number of EPCs observed on the surface of the RGD-modified hydrogel was higher compared to the non-RGD-modified one. In addition, EPCs dispersed radially in the modified hydrogels. The results indicate that the RGD peptide significantly increases the adhesion and proliferation of EPCs [79]. Therefore, the natural presentation of the motif in the DEM structures is pivotal in directing cell adhesion and migration in the structures.

The biomechanical properties of native ECM are also known to influence cell adhesion, proliferation, migration, and differentiation. Mechanical forces such as tissue stiffness affect cell responses by transmitting through mechanotransduction pathways and sensing by integrin receptors [13]. Integrin receptors function by the bidirectional transducing of biochemical signals and mechanical force across the plasma membrane. The receptors attach to ECM ligands by their extracellular domains and engage with intracellular signaling pathways and cytoskeletal proteins by their cytoplasmic tails [80]. Complex structural rearrangements enable integrins to engage different ligands and activate diverse downstream signaling networks [81]. Importantly, the dynamic and mechanosensitive signaling network actively controls cell shape and force generation and propels adherent cell self-renewal and survival, proliferation, and differentiation [15]. Regarding the role of biomechanical cues in the cell fate, it is shown that the polyacrylamide gels that were functionalized with DAT promoted adipogenic differentiation and up-regulated adipogenic markers even in the absence of exogenous adipogenic growth factors only when they mimicked native adipose tissue stiffness (i.e., 2 to 4 kPa) [82,83]. On the contrary, as the stiffness increased, ASCs became more spread and lost their typical rounded morphology, and did not up-regulate adipogenic markers [83] (Fig. S1). Furthermore, a study revealed that adipogenic differentiation was enhanced in the microporous DAT foams compared to bead foams, potentially due to the matched stiffness of the DAT foams to the native tissue. In vivo assessment demonstrated that the DAT foams were well tolerated and integrated into the host tissues, and supported angiogenesis and adipogenesis [84].

Structural properties are the third feature of DEMs that influence infiltrated cells’ behavior, such as scaffold porosity that facilitates nutrient and oxygen diffusion and modulates cell–cell and cell–ECM interaction. Such structural properties dictate the surface area available for cell–ECM and cell–cell contacts [13]. It is indicated that the dense ECM structure within the intact decellularized tissue matrix limits cell infiltration and hinders stem cells from localizing within the central regions of the scaffolds. Accordingly, manufacturing a 3D scaffold consisted of ECM-derived bead foams seeded with ASCs resulted in a high density of viable cells distributed throughout the constructs and resulted in robust adipogenesis and cellular infiltration [85]. A tissue construct using DAT bioink and ASCs was also developed [86,87]. The printed DAT constructs, which had less dense structure, expressed adipogenic genes more intensely than nonprinted DAT gel. The constructs also supported positive tissue infiltration, constructive tissue remodeling, and adipose tissue formation in mice subcutaneous models [87]. Moreover, a study that compared an injectable ADM and a sheet-type ADM showed that injectable ADM, which was less dense, supported more intense fibroblast proliferation and tissue integration along with comparable volume retention [88]. Figure 4 provides a schematic of the mechanisms underlying cell interactions with decellularized scaffolds.

## DEMs’ Current Applications in Clinical Studies

Reconstructive and aesthetic surgery still faces challenges with filling soft tissue defects. Whether caused by trauma, congenital anomalies, or iatrogenic factors, these defects can significantly impact patients’ psychological well-being and quality of life. Autologous tissue flaps are the source that is usually utilized by plastic surgeons for the reconstruction of soft tissue defects [89]. The surgical operation is nevertheless challenging due to the uncertainty of microsurgical tissue transfer and the possibility of donor site morbidity. Fat grafting is an effective and safe option to correct small- and mid-sized volume deficiencies. However, clinical challenges include donor site morbidity, graft loss, calcification, and oil cysts [3].

Alternatively, common implantable biomaterials include hyaluronic acid, collagen, and polymethyl methacrylate. While these biomaterials offer easy access and minimal injury to the donor area, they may lead to foreign body reactions, inflammation, and rapid absorption of the biomaterial [4]. The use of DEM scaffolds has been translated to the clinic with varying levels of success. These biomaterials have a growing application in facial filling, breast reconstruction, penile augmentation, and rhinoplasty and are derived from different sources, including human, porcine, and bovine sources.

This section reviews the recent experiences in applying DEMs as soft tissue fillers and their clinical outcomes and adverse complications. Notably, we only reviewed some most recent and robust studies in the section, chosen out of an abundant number of clinical studies. Table 2 presents studies on DEM trade products across a broad spectrum of clinical applications as soft tissue fillers.

**Table 2.** Examples of Clinically Used Decellularized Extracellular Matrix Products as Soft Tissue Fillers^*^

Product name, origin	Manufacturer	Fabricated forms	Clinical applications	Manufacturing Country	References
**Acellular dermal matrix (ADM)**					
BellaDerm®, Human	Musculoskeletal Transplant Foundation	Sheet	Facial filler	USA	[1]
Strattice™, Porcine	Lifecell	Sheet	Breast reconstruction	USA	[2,3]
AlloDerm®, Human	Lifecell	Sheet	Breast reconstruction, Rhinoplasty, Penile augmentation, Facial filler	USA	[2,4–13]
SurgiMend®, Bovine	Integra LifeSciences	Sheet	Breast reconstruction, Penile augmentation	USA	[3,4,6,14]
FlexHD®, Human	MTF Biologics	Sheet	Breast reconstruction	USA	[2,15–17]
Cortiva™, Human	RTI Surgical	Sheet	Breast reconstruction	USA	[18]
DermACELL®, Human	LifeNet Health	Sheet	Breast reconstruction	USA	[19]
MegaDerm™, Human	L&C BIO	Sheet	Breast reconstruction, Rhinoplasty, Penile augmentation	China	[20–23]
CryoDerm, Human	CGBio	Sheet	Breast reconstruction	South Korea	[22]
InteXen®, Porcine	American Medical Systems	Sheet	Penile augmentation	USA	[12]
J-1 ADM, Human	Beijing J.Y. Life Tissue Engineering	Sheet	Penile augmentation	China	[24]
BellaGen™, Human	Hans Biomed	Powder suspension	Penile augmentation	South Korea	[25]
MegaFill®, Human	L&C BIO	Powder suspension	Penile augmentation, Facial Filler	China	[26,27]
Cymetra, Human	Lifecell	Powder suspension	Facial filler	USA	[28]
**Decellularized adipose tissue matrix (DAT)**					
Renuva®, Human	MTF Biologics	Powder suspension	Facial filler	USA	[29]
**Small intestinal submucosa (SIS) matrix**					
Surgisis®, Porcine	Cook Biotech Inc	Sheet	Facial filler	USA	[30,31]

* The DEM products were limited to those clinically applied as soft tissue fillers and results were reported in research papers. Furthermore, the products are not limited to papers referenced here.

### Facial filler

Aged skin characteristically displays a substantial loss of elasticity, sagging, the appearance of wrinkles, and deepening of tissue folds. Skin volume and strength are reduced in response to a reduction in collagen content, causing wrinkles to appear. In the past decade, hyaluronic acid fillers and autologous fat transfer have provided an attractive nonsurgical alternative to decrease skin wrinkles by compensating for the aging-associated loss of the dermal ECM volume [90]. In addition to cosmetic concerns, these fillers are also tried for patients suffering from congenital defects, trauma, or surgical resections [4].

In recent years, DEM have attracted growing attention to be used as facial fillers. The potential of DAT in developing fat tissue after subcutaneous injection is clinically documented, and the material has been used as a facial filler. Injecting DAT suspension into the subcutaneous wrist dorsum or abdominal tissue showed a maintained soft tissue volume with a texture consistent with subcutaneous adipose, and no severe adverse event [41]. Studies have also shown an increase in the infiltrating adipocytes over time concurrent with the turnover of the injected DATs, which indicates the regenerative nature of the allogeneic matrix [91]. Furthermore, the formation of blood vessels and the migration of ASCs and immune populations indicated ongoing tissue remodeling and long-term tissue replacement [45].

In a phase 1 clinical study on healthy volunteers, cellular migration from the host tissue into the implant was evident at the host–implant boundary and increased with the duration of implantation [45]. As shown in Fig. S2, at 18 weeks after injection, new blood vessels are formed at the edge of the implant, shown by the infiltration of CD31^+^ vascular ECs (Fig. S2). Perivascular/adipose stem cells (CD34^+^) also infiltrated around and within the implant. By this time, CD4^+^ T cells dominated within the T cell population recruited by the scaffold. Figure S2 demonstrates how in situ recellularization, constructive immune response (indicated by CD4^+^ T cell dominance), and angiogenesis (indicated by CD31^+^ cells infiltration) collaboratively contribute to the integration of DEM implants. Another study that implemented the DAT in correcting temple atrophy showed that DAT injection bilaterally into the atrophic temples provided at least 6 months of volume retention and promoted autologous fat formation and tissue remodeling [92]. The histopathological results on biopsy specimens were also consistent with adipogenesis and vascular growth at the temples.

ADM is another biomaterial studied for facial augmentation, which has been used for both cosmetic purposes and correcting congenital defects. In a study, the effects of micronized ADM in rejuvenating the aging and atrophic lip were evaluated, and patients treated with micronized ADM compared to those treated with collagen filler had a more durable volume expansion and better aesthetic results after 12 months [93]. Micronized ADM filler was also applied to correct the atrophic defect of linear morphea (sclerotic skin disease) and showed favorable aesthetic outcomes and long-lasting volume augmentation [94]. Another clinical study evaluated the safety and efficacy of a hyaluronic acid filler mixed with micronized cross-linked ADM in patients with human immunodeficiency virus (HIV)-associated facial lipoatrophy [95]. All patients showed a significant improvement and no adverse events were reported, except for one case of transient subcutaneous nodule formation. The result could be attributed to the fact that ADM contains the necessary extracellular components for cell integration and drive long-term ECM remodeling by increasing the expressions of type I collagen, MMP-1, MMP-2, and TGF-β for up to 6 months [96].

Sheet-type ADM has also been used to correct facial volume defects. A study inserted a small strip of ADM underneath the deep glabellar rhytides wrinkle line, which is hard to be corrected by filler injection, and the deep rhytides showed significant and long-lasting improvement after the procedure [97]. ADM sheet was also applied in correcting tear trough deformity by suturing the tissue to the periosteum and fixing to the orbital septum and showed favorable volume improvement for patients. The study demonstrated regeneration of blood vessels and reorganization of collagen fibers in the implant site 1 year after the operation [98].

### Breast reconstruction

Breast cancer is the world’s most prevalent cancer, having a lifetime risk of 10 to 14% among women. Up to 70% of newly diagnosed patients undergo mastectomy as the choice of treatment [99]. ADM was first introduced in 2005 to recreate the inferior pole of the pectoralis major muscle, which shortens the surgery time. Since then, the use of ADM in breast reconstruction surgeries has become common and employed in up to 56% of all tissue expander and implant-based breast reconstructions [99]. These biological materials provide structural strength and bulk, as well as relatively rapid vascular ingrowth, and serve as a scaffold for tissue regeneration. The most common application of ADM involves covering the inferior pole of the implant in immediate post-mastectomy prosthesis-based breast reconstructions. Surgical techniques utilizing ADM are various; however, in the setting of immediate or delayed breast reconstruction, the ADM sheet is used as an extension of the pectoralis major, which is sewn from the inferior edge of the muscle to the inframammary fold and completely covers the implant [100]. Therefore, ADM provides an alternative soft tissue option for surgeons to recruit adjacent muscles and fascia.

Despite individual reports of complications, aggregate data suggest that they are relatively rare [10]. At both short- and long-term follow-ups, ADMs demonstrated excellent integration into host tissues with neovascularization, cell repopulation, and absence of inflammatory cells [101,102]. ADMs facilitated symmetrical coverage and enhanced the aesthetic result, decreased pain associated with pectoralis muscle mobilization, and reduced scarring [103,104]. The ADM surface was more resistant to the formation of capsules and scarring, which was attributed to the decreased inflammatory response and exhibition of enhanced elasticity upon revascularization [105,106]. The integration of ADMs has been shown to reduce the risk of infection, shorten the duration of tissue drainage, and decrease the length of hospital stays [107].

Although post-mastectomy radiation therapy typically increases the risk of surgical complications in autologous reconstruction techniques, using ADM in immediate breast reconstruction followed by post-mastectomy radiation therapy has been shown to yield good aesthetic and reconstructive outcomes [108,109]. Regarding procedure costs, ADMs are associated with higher short-term expenses for patients; however, the 2-year postoperative costs are lower [110].

However, the ultimate outcome of using ADM implants in breast reconstruction depends on many factors. Studies have shown that size and shape [111], thickness [112], manufacturer [113], and species origin [114,115] result in a change in the incidence of complications, ultimate shape of the breast, implant loss, and reoperation rate. For instance, it is shown that the use of thick ADM implants (>1.2 mm) compared to thin implants (<1.2 mm) results in higher rates of necrosis, seroma, infection, and a greater need for drainage 2 weeks postoperatively. Thicker ADMs may have less neovascularization, which may be the reason for more complications [112]. Different products may also perform differently. In comparative studies on ADM outcomes in breast reconstruction, FlexHD caused more infections than AlloDerm and Cortiva, while all of the products are human-derived ADMs [113]. Studies that compared ADMs of different origin species indicated that bovine-derived ADM may be more suitable for breast reconstruction than human-derived ADM and porcine-derived ADM. SurgiMend (bovine derivative) and AlloDerm (human derivative) showed similar rates of major early complications, but AlloDerm had a significantly higher rate of seroma [114]. Additionally, the clinical outcomes of SurgiMend were better than Strattic (porcine derivative), in factors including implant loss and reoperation rates [115]. ADM procedures are also known to be affected by patient lifestyle factors. Smoking, chemotherapy, radiation, obesity, and diabetes mellitus are associated with an increased risk of complications and implant failure [102,112,116–118]. Further research is needed to delineate the mechanisms underlying different outcomes shown in the clinical studies.

### Penile girth and length enhancement

Penile length enhancement may be performed for aesthetic purposes or to correct congenital diseases, such as buried penis. An ideal treatment to increase penile length should involve minimal incisions, limited scarring, and no interference with erectile function [119]. The experimental nature of this surgery and the complexity of the patient’s psychological status have made the selection of the surgical technique highly controversial, and no methods have been unanimously approved.

As a treatment option, ADM implants have been used for both penile length augmentation. The outcomes of penile length augmentation with ADM implants have been satisfactory, with rare complications. ADM implant surgery benefited from reducing necrosis risk and surgery time and allowing for a more subtle incision [119,120]. Additionally, a study involving suprapubic liposuction, penile suspensory ligament release, and the insertion of a folded ADM between the corpora cavernosa and pubic symphysis reported an 80% increase in penile length [121].

Regarding penile girth enhancement using ADM implants, there are controversial reports of the efficacy and safety of the procedures. While some studies report a substantial rate and severity of complications [122], other studies indicate that these procedures can be safely performed with standard surgical techniques, rigorous postoperative care, and sufficient notification to the patients [123]. In a study, porcine ADM was placed circumferentially between the cavernous and spongious bodies and showed successive increases in penile circumference with no major complications [119]. Moreover, penile girth enlargement by ADMs may improve erectile function and reduce premature ejaculation among penile-augmented patients [124].

### Rhinoplasty

Given that Asian noses tend to be small and flat, rhinoplasty procedures frequently focus on dorsal augmentation and tip projection. As a result, implant materials are commonly used in rhinoplasty for Asian noses. Although autogenous implants represent the gold standard for this purpose, they suffer from the risk of donor site morbidity with limited amounts of available tissue and unpredictable resorption rates. Conversely, alloplastic materials, especially silicone, come with disadvantages, including a higher risk of implant extrusion, mobility, and infection. Recently, ADMs have been introduced for dorsal augmentation benefiting from ECM precipitation and enhanced tissue regeneration. Cross-linked human ADM demonstrated relatively high biocompatibility and a low infection rate. It also showed maintained structural integrity and durability after implantation [125]. ADM has also been used for nose irregularities enhancement. A study reported the use of ADM for palpable or visible bony irregularities on the nasal dorsum as a result of previous rhinoplasty or trauma, and palpable bony irregularities were successfully covered by an ADM sheet [126].

A retrospective cohort study enrolled 145 primary or revision dorsal augmentation rhinoplasty patients operated by a single surgeon during a 65-month interval. The satisfaction rates of the surgeon and patients were comparable between the primary and revision surgery groups. Microscopic examination of the removed ADMs revealed abundant collagen tissue and a newly formed vascular network without any signs of foreign body reaction. The results suggested that ADM can be utilized in both primary and revision dorsal augmentation rhinoplasty, with minimal complications [127]. Another study demonstrated that the use of ADM achieved a pooled success rate of 96.0%, with a minimal re-operation rate of 2.0% and a total complication rate of 2.0%. Therefore, ADM can be a suitable alternative for autografts in nasal augmentation/reconstruction rhinoplasty surgery [128].

## Bottlenecks and Challenges in Translating DEM Fillers into Clinical Applications

DEM products offer the opportunity to address a wide range of unmet clinical needs. However, there are challenges in meeting clinical demands that need to be considered. In this context, less invasive delivery roots should be examined for DEM implantation, the biodegradability of DEM structures should be tuned, and a deeper understanding should be developed regarding the cellular response to the biomaterials and the role of specific ECM components in the regeneration process.

As mentioned, DEM scaffolds are clinically used in various applications, from breast reconstruction to facial fillers. Accordingly, procedures implanting DEMs into host tissues can vary from invasive surgeries to minimally invasive injections. In some applications, such as lip and cheek augmentation, physicians would need injectable materials able to pass from thin needles. Hyaluronic acid fillers are typically injected through 27- to 30-gauge needles depending on filler particle size, concentration, and cross-linking degree, which determines the viscosity or thickness of the gel [129]. Injecting fillers through higher gauge needles could potentially enhance the injection process by making biomaterial delivery and placement more accurate. Such injections result in a more balanced and symmetrical outcome, less injection pain, less bleeding/bruising, and higher patient satisfaction [129]. However, DEM fillers have never been injected through needles with higher than 25 gauge in previous studies [70,92,130], and there is a lack of studies to assess the feasibility and clinical outcomes of injecting DEMs through thinner needles. Some studies also used graft implants as facial filler material. For instance, acellular freeze-dried SIS in the form of graft strands was implanted into patients’ lips and nasolabial folds [131,132]. In these procedures, incisions were created for the entrance and exit sites of the trocar at the limits of the lip or nasolabial folds. The trocar was then inserted into one incision site and passed through the full length of the area being treated to the other incision site. Although the authors mentioned that patients tolerated the procedure well, the procedure invasiveness would be a barrier to patient satisfaction in wide clinical applications. Therefore, factors that enable biomaterials to pass through thinner gauge needles and the clinical outcome of applying the biomaterials need to be elucidated.

The second noteworthy challenge in the development of DEM filler materials is their biodegradability, which might cause long-term degradation and unsatisfactory clinical outcomes. ECM degradation would shrink the injected or implanted filler volume and mandate the filler replacement, which is cost-bearing, frustrating, and invasive. There is extensive research on enhancing the filler’s biodegradability. The metalloproteinase family of proteases, particularly MMPs, are significant enzymes involved in ECM remodeling, which are secreted from infiltrating cells. Serine proteinases such as plasmin and cathepsin G also participate in degrading ECM protein components [133]. Cross-linking of ECM components, as a method to enhance DEM fillers’ biodegradability, can increase resistance to enzymatic degradation but may affect angiogenesis, tissue formation, and inflammatory response [96]. Common agents used for cross-linking collagen-based biomaterials include glutaraldehyde, genipin, carbodiimide, transglutaminase, and reduced riboflavin [134]. It is shown that cross-linkers can improve the mechanical properties and enzymatic resistance of ECM without any detrimental effect on cells’ viability [5].

However, there are controversial results on the effect of cross-linking on the immune system reaction. Some studies have shown that chemical cross-linking agents can elicit an inflammatory immune response by activating macrophages, increasing the proinflammatory cytokine release, and inclining the macrophage population toward the M1 phenotype [135], whereas other studies indicate that cross-linking is a modulator of the immune response, even when glutaraldehyde is used, which is an immunogenic agent [136]. As a matter of fact, glutaraldehyde has been utilized in many clinically approved ECM-based applications to improve the mechanical properties and lifetime of the biomaterials. These clinical applications include preserving and cross-linking bioprosthetic heart valves, patch materials, and decellularized organs [137–139]. The adverse effect of glutaraldehyde has been documented by some studies in causing calcification, pro-inflammatory effects, and cytotoxicity [140]. Therefore, the trend in cross-linking biomaterials is toward utilizing finely titrated glutaraldehyde cross-linking protocols or utilizing less toxic cross-linking agents such as genipin [138,140]. Further studies are needed to evaluate safe and efficient cross-linking agents, methods, and doses to tune the biodegradability of DEM fillers.

Alternatively, the biodegradability of the fillers may be enhanced by mixing ECM powders with synthetic or natural polymers [141]. Polymers such as PLGA (poly d, l-lactide-co-glycolide), alginate, silk fibroin, and chitosan have been mixed with DEMs to tune their properties and degradation rates [9,141]. These polymers are also shown to improve tissue mechanical and antimicrobial properties, as well as provide additional inherent bioactive motifs [9]. Hybrid composites containing synthetic materials and natural ECMs may retain the benefits of both materials that are reproducible mechanical properties of polymers and good biocompatibility of DEMs [141]. In this regard, a photo-cross-linkable injectable prepolymer was produced to encapsulate human ASCs. The bioscaffold incorporated DAT and photo-cross-linkable methacrylated chondroitin sulfate delivery vehicles. The encapsulation strategy provided an effective method of efficient and uniform cell delivery, which could be adapted as a minimally invasive in situ approach. In the gels that are produced after photo-cross-linking, DAT acted as a cell-supportive matrix that enhanced ASC viability, retention, and adipogenesis [69].

Adding matrix metalloproteinase inhibitors (MMPIs), such as doxycycline, as a third method to enhance DEM fillers’ biodegradability, has been shown to modulate the degradation rate of DEMs. MMPIs are shown to be effective in reducing degradation without affecting material biocompatibility or mechanical properties [134]. In a recent study conducted in our laboratory, we demonstrated that the application of doxycycline as an MMP inhibitor leads to higher preservation of collagen, glycosaminoglycans, and growth factor contents of the decellularized matrix during decellularization [142]. Notably, the bioscaffolds were biocompatible and supported cell viability, proliferation, and functionality in vitro. However, studies on the application of MMPIs to modulate the degradation rate of filler biomaterials are scarce, and more studies are needed to delineate the effect of MMPIs such as clodronate and alendronate, which are suppressors of macrophage lineage, on the bioactivity of the DEM structures. Moreover, the consequences of interfering with MMPs as substantial proteins in the angiogenesis process on the outcome of filling procedures are not evident.

As the third bottleneck, there is still much unknown on the cellular and molecular response to the biomaterials, the role of specific ECM components in tissue regeneration, and the interaction between the immune system and DEMs in tissue restoration. Characterizing the mechanical, physical, and biomechanical host responses to biomaterials is a prerequisite for the future development of DEM fillers [9]. Although ECM scaffolds have been used in clinical settings for many years, there is still a lack of comprehensive understanding of ECM scaffolds, their mechanisms of action, and their immunological impact on humans. Therefore, further biological insights into spatiotemporal host response to the biomaterials are needed.

## Future Prospective

This review initially discussed the roles of DAMPs, neutrophils, macrophages and their polarization, and T cells in the host immune response to DEM biomaterials. Then, we noted the ECM components and host responses that take part in the angiogenesis and vasculogenesis of DEMs. The compositional, biomechanical, and structural features of DEM scaffolds that guide cell adhesion, proliferation, and differentiation were also mentioned. Moreover, the current applications of DEMs as biomaterials for facial filling, breast reconstruction, penile augmentation, and rhinoplasty were reviewed. Furthermore, some challenges in translating DEM fillers into clinical applications were discussed, comprising the need for minimizing the invasiveness of the implanting procedures, enhancing biomaterials’ biodegradability, and fundamental requirements for further characterizing the host response to DEMs. Altogether, studies show promising results confirming that the fillers can cause a long-lasting volume enhancement and restore and support angiogenesis, fibroblast activation, cell infiltration, and differentiation, with minimal risk of inflammation.

In our opinion, many nascent research areas in the field warrant further attention. For instance, various tissue sources for decellularized biomaterials with broad research or even clinical background have not been tested as filler material in the clinical setting including but not limited to the amniotic membrane and cartilage. The amniotic membrane is a homologous, readily available, and inexpensive biological material with no in vivo rejection, making it an important source of scaffolding material [143,144]. The amniotic membrane contains an array of growth factors, cytokines, and chemokines and can promote the proliferation and migration of mesenchymal stem cells and ASCs [8]. Although no studies were found on the use of decellularized amniotic membrane as a soft tissue filler, the ReNu cryopreserved amniotic suspension allograft, containing both ECM components and amniotic fluid cells, has been used to correct mid-face volume loss and signs of aging [145]. The amniotic allograft caused superior improvement in the mid-face volume when compared with that of platelet-rich plasma.

The potential of using a processed amniotic membrane-derived product (manufactured by PURION process), consisting of cellular human amnion, intermediate layer, and chorion membrane, was investigated in soft tissue-filling procedures. The study showed that extracts that are derived from the product have a dose-dependent mitogenic effect on human dermal fibroblasts and promote greater cell migration. To examine the cellular response to the biomaterial in athymic nude mice model, 1 cm × 1 cm grafts were implanted subcutaneously. Histological analysis showed progressive remodeling of the biomaterial throughout the 4-week study. Host cell infiltration and ingrowth was evident 2 and 4 weeks after implantation. The scaffold showed reorganization, characterized by resorption and deposition of the collagenous matrix, and demonstrated neovascularization at week 4. Additionally, histological assessment showed no major inflammatory reaction [146]. Another study investigated the efficacy of folded and injectable cellularized amniotic membranes as soft tissue fillers [143]. Multilayer amniotic membranes were transplanted to the subcutaneous tunnels created in the rat’s back. Alternatively, the amniotic membrane was transformed into an injectable material and was inoculated transcutaneously under the rat’s skin. Both multilayer amniotic membrane grafts and injectable amniotic membranes showed long-term maintenance of their volume and limited foreign body reactions, fibrosis, and necrosis. Accordingly, an unpublished study conducted in our laboratory showed that the powder suspension of the cross-linked decellularized human amniotic membrane was able to preserve more than 50% of the measured volume augmentation 4 weeks after injection into the subcutaneous tissue of the guinea pig model. Amniotic/chorionic membrane analysis has revealed that inhibitors of MMPs overwhelmingly outnumbered the MMP enzymes by an overall molar ratio of 28:1, and the MMPs mainly exist either in their latent form or complexed with inhibitors [8]. The finding recommends that decellularized amniotic tissue can largely be spared from MMP-mediated degradation in soft tissues and might have longer volume retention.

The use of autologous or homologous cartilage in rhinoplasty is increasingly common because of its low absorption, low infection, and shape-maintaining ability. However, due to the coarse nature of cartilage particles and the time-consuming, invasive process required, there has been a demand for ready-made and commercialized materials derived from cartilage [147]. Along with a wide range of applications in the reconstruction of complex cartilage defects, cartilage provides a good scaffold for soft tissue augmentation. Cartilage matrix has a limited vascular supply and lymphatic influence, which gives the tissue immunological advantages compared to other tissues, especially when the origin is xenogenic [148]. The lyophilized acellular articular cartilage derived from cadaveric human cartilage tissue was produced in the injectable powdered form and injected subcutaneously into the mice model [147]. The volume retention of the lyophilized articular cartilage matrix was comparable to hyaluronic acid and ADM. Moreover, it exhibited faster bruise resolution in safety tests. Articular tissue in powder formulation was also injected subcutaneously into the rat model and compared with polymethylmethacrylate and calcium hydroxyapatite fillers. There was lesser infiltration of giant cells in the injection site in the decellularized cartilage matrix group. Thus, decellularized cartilage matrices may have a distinct advantage in terms of biocompatibility compared with other commercial injectable long-lasting fillers. Further research focusing on the integrating and angiogenic properties of the material and its interplay with the immune system is needed [148]. Considering that numerous products of cartilage matrix, such as ROKIT Bio-filler [149], have solid evidence of safety in the treatment of cartilage defects and osteoarthritis, broadening their application to soft tissue augmentation is on the horizon.

As another naive research area, the use of DEM filler components as bulking agents can be suggested as a convenient treatment for stress urinary incontinence, since materials with fundamentally similar structures to DEMs showed promising results in this regard. A study developed a bioactive injectable bulking agent that consists of Permacol, which is made of collagen, and recombinant insulin-like growth factor-1-conjugated fibrin micro-beads and injected the material into the bladder of the rabbit model of stress urinary incontinence. After 3 months, the conjugated product showed no adverse effect, and smooth muscle tissue-like formation was observed within the injected material [150]. Another injectable bulking agent was developed through decellularizing ECM fragments produced by cultured ASC sheets. The material was implanted in the stress urinary incontinence rat model. Improvement of urethral sphincter function was observed and new smooth muscle tissue had formed around the ASC ECM fragments [151]. These results indicate that the future application of DEM fillers as bulking agents may be a minimally invasive approach for treating urinary incontinence.

Another research area warranting further investigation involves strategies to enhance angiogenesis and vasculogenesis in biomaterials. To our knowledge, these strategies have not yet been applied to DEM fillers. The development of a functional vascular network within decellularized constructs is essential for their integration following in vivo implantation [152]. Filler biomaterials that cannot induce neovascularization would fail to support cell infiltration and tissue integration [153,154]. Research demonstrates that combining an acellular blood vessel matrix with other biomaterials can significantly enhance angiogenic properties. Acellular blood vessel matrix hydrogels have revealed their pro-angiogenic effects in vitro and their therapeutic effects in the skin flap model [155]. Plates coated with blood vessel matrix hydrogel promoted tube formation by HUVECs. Injecting the hydrogel into skin flaps enhanced skin flap survival and increased blood perfusion and capillary density. In another study, scaffolds composed of varying ratios of silk fibroin and acellular blood vessel matrix were fabricated using the freeze-drying method [154]. The acellular blood vessel matrix gel increased the number of vascular loops and CD34-positive ECs than the pure silk fibroin control scaffold after subcutaneous implantation in rats. Thus, combining acellular blood vessel matrix with DEM materials promises to enhance filler integration with host tissues. Another strategy to enhance blood and nutrient transportation to DEM biomaterials is to use progenitor cells such as EPCs and HUVECs for the rapid formation of blood vessel networks. It is shown that EPCs can induce in situ vasculogenesis within 7 days in the mice model and form patent perfused blood vessels in vitro [156]. Another study that injected an ECM-based matrix seeded with EPCs into the mice model showed that the EPCs formed functional blood vessel formation after 7 days [157]. Staining of vessels for α-smooth muscle actin (αSMA), which is expressed by perivascular cells but not ECs, showed that host perivascular cells are recruited to form functional blood vessels when EPCs were used [156]. As another choice for vasculogenesis induction in biomaterials, a study showed that human ASC and HUVEC cocultures were able to induce vasculogenesis when subcutaneously injected into the mice. A stable neovasculature, characterized by perfusion with erythrocytes, was formed, and co-implanted ASCs displayed perivascular properties by stabilizing these neovessels [158]. Exploiting the promising research on this matter, the induction of in situ vascularization in DEM fillers, whether by adding progenitor cells or recruiting host progenitor cells to the constructs, can lead to the development of long-lasting filler materials with high host integration capacities.

Like any other material, native DEMs face some limitations, including batch-to-batch variability across different donors, low reproducibility, and limited control over construct properties, such as porosity, stiffness, mechanical stability, and degradability [135]. Hence, native DEMs are largely restricted to the source tissue features. In soft tissue filling using DEM biomaterials in the preclinical settings, the DEMs are usually further processed, which allows obtaining different products with specific characteristics that possess fine-tuned particular properties for the desired applications. Powder suspension in cell culture medium, injectable hydrogels, porous and bead foams, 3D printed bioinks, and scaffold sheets are the most widely used formulations of DEMs for soft tissue filling in the preclinical setting, each of which has their own limitations and strengths. However, in clinical settings, the application of DEM biomaterials as soft tissue fillers has been limited to scaffold sheets and powder suspension. Although the application of off-the-shelf commercially available DEMs increased and varied over the past 2 decades, the commercial products that have been used for soft tissue augmentation were limited to the sheets and powder suspension formulations (Table 2). Therefore, further research on the translation of other DEM formulations into clinical studies would be essential.

## Conclusion

In conclusion, DEM-based fillers have demonstrated considerable promise in regenerative medicine by facilitating long-lasting volume enhancement and actively supporting processes such as angiogenesis, fibroblast activation, and cell infiltration and differentiation. Furthermore, innovative strategies employing acellular blood vessel matrices and progenitor cells like EPCs and HUVECs have laid the groundwork for the development of fillers that not only integrate with host tissues but also foster the rapid formation of functional vascular network. Such progress highlights the potential of DEM fillers to revolutionize soft tissue augmentation and underscores their significance in enhancing tissue viability and integration after implantation.

However, the journey from laboratory to clinic faces significant challenges, including the need for more comprehensive clinical studies with extended follow-up periods to evaluate the long-term safety and efficacy of DEM fillers. Despite promising initial results, the current body of evidence is insufficient for widespread clinical adoption, highlighting the urgent need for robust, large-scale studies. Addressing these challenges is essential for advancing DEM technologies and providing a solid evidence base to support their clinical application, thereby ensuring physicians and patients.
