# Supplementary figures and images for: Decellularized Extracellular Matrix Scaffolds for Soft Tissue Augmentation: From Host–Scaffold Interactions to Bottlenecks in Clinical Translation
Source: Biomater Res. 2024 Sep 6;28:0071. doi: 10.34133/bmr.0071 (PMC11378302; doi:10.34133/bmr.0071)

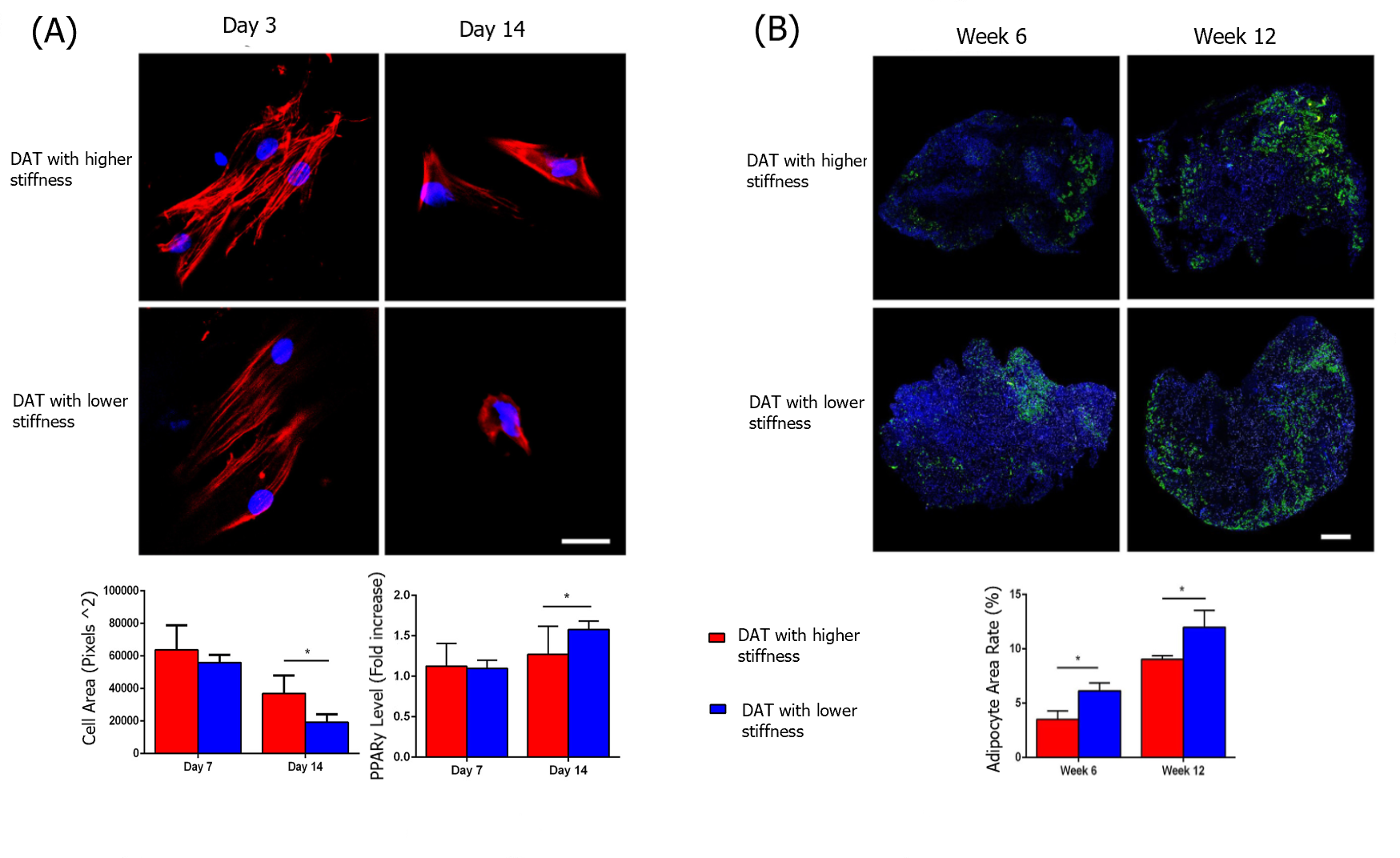

Supplement: Supplementary 1 — Figs. S1 and S2 [file bmr.0071.f1.zip › Supplementary Figure 1.png]

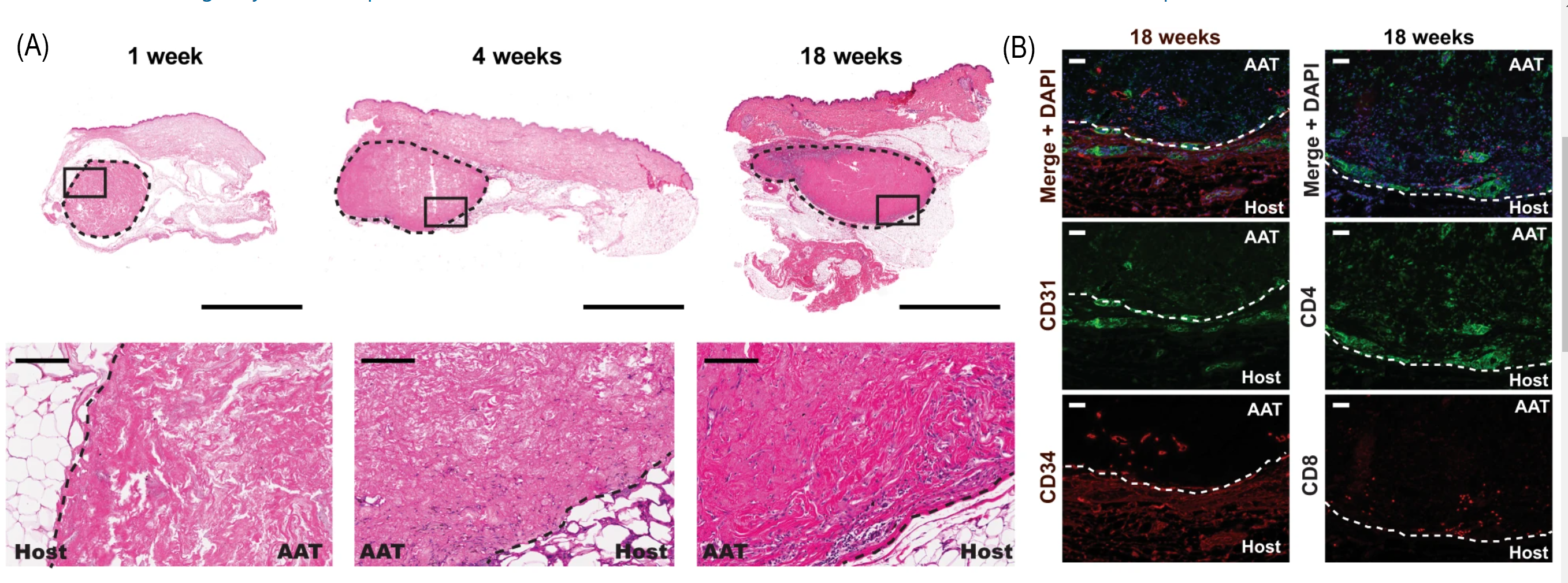

Supplement: Supplementary 1 — Figs. S1 and S2 [file bmr.0071.f1.zip › Supplementary Figure 2.png]
